# An integrated Asian human SNV and indel benchmark established using multiple sequencing methods

**DOI:** 10.1038/s41598-020-66605-6

**Published:** 2020-06-17

**Authors:** Chuanfeng Huang, Libin Shao, Shoufang Qu, Junhua Rao, Tao Cheng, Zhisheng Cao, Sanyang Liu, Jie Hu, Xinming Liang, Ling Shang, Yangyi Chen, Zhikun Liang, Jiezhong Zhang, Peipei Chen, Donghong Luo, Anna Zhu, Ting Yu, Wenxin Zhang, Guangyi Fan, Fang Chen, Jie Huang

**Affiliations:** 1 National Institutes for food and drug Control (NIFDC), No.2, Tiantan Xili Dongcheng District, Beijing, 10050 P. R. China; 2 BGI-Qingdao, BGI-Shenzhen, Qingdao, Shandong 266555 P. R. China; 3 MGI, BGI-Shenzhen, Shenzhen, Guangdong, 518083 P. R. China; 4 BerryGenomics Co., Ltd. Building #5, 4 Science Park Road, ZGC Life Science Park, Beijing, 102200 P. R. China; 5 Tianjin Novogene Bioinformatic Technology Co., Ltd. Entrepreneurial Headquarters Base B07-B09, Wuqing Development Zone, Tianjin, 301700 P. R. China; 6 Annoroad Gene Technology, Building B1, Yard 88, kechuang 6Rd, Beijing Economic-Technological Development Area, Beijing, 102200 P. R. China; 7 CapitalBio Genomics Co., Ltd., Building 11, GuanTai Biotechnology Cooperation Incubation Center, No.1, Taoyuan Road, Songshan Lake Hi-Tech Industrial Development Zone, Dongguan, Guangdong, 523808 P. R. China; 8 Guangzhou Daruia Biotechnology Co. Ltd., 5 buildings No. 11 Nanxiang Third Road, Science City, Luogang District, Guangzhou, Guangdong, 510663 P. R. China; 9 BGI-Shenzhen, Shenzhen, Guangdong, 518083 P. R. China; 10 China National GeneBank, BGI-Shenzhen, Shenzhen, Guangdong 518120 P.R. China

**Keywords:** Rare variants, Next-generation sequencing

## Abstract

Sequencing technologies have been rapidly developed recently, leading to the breakthrough of sequencing-based clinical diagnosis, but accurate and complete genome variation benchmark would be required for further assessment of precision medicine applications. Despite the human cell line of NA12878 has been successfully developed to be a variation benchmark, population-specific variation benchmark is still lacking. Here, we established an Asian human variation benchmark by constructing and sequencing a stabilized cell line of a Chinese Han volunteer. By using seven different sequencing strategies, we obtained ~3.88 Tb clean data from different laboratories, hoping to reach the point of high sequencing depth and accurate variation detection. Through the combination of variations identified from different sequencing strategies and different analysis pipelines, we identified 3.35 million SNVs and 348.65 thousand indels, which were well supported by our sequencing data and passed our strict quality control, thus should be high confidence variation benchmark. Besides, we also detected 5,913 high-quality SNVs which had 969 sites were novel and  located in the high homologous regions supported by long-range information in both the co-barcoding single tube Long Fragment Read (stLFR) data and PacBio HiFi CCS data. Furthermore, by using the long reads data (stLFR and HiFi CCS), we were able to phase more than 99% heterozygous SNVs, which helps to improve the benchmark to be haplotype level. Our study provided comprehensive sequencing data as well as the integrated variation benchmark of an Asian derived cell line, which would be valuable for future sequencing-based clinical development.

## Introduction

Sequencing technologies have been revolutionized in recent decades with the sequencing cost to have been dramatically reduced^1,2^. Thus, human genomes are now sequenced not only for research purposes^3^ but also for clinical applications^4^. Especially more recently, large-scale population sequencing projects^5–8^ have been proposed to fulfill precision medicine and reveal genomic mechanisms of more diseases. With the rapid upgrade of sequencing techlogies, we are anticipating a routine usage of human genome sequencing in daily healthcare in the near future. Considering its wide applications, we need to carefully assess different sequencing technologies for ensuring safety and accuracy, as well as accelerating the sequencing-based applications. Accordingly, a human genome variation benchmark is required. Currently, a standard variation dataset of NA12878, a cell line of Caucasian origin, has been established^9^. Significant insights have been gained from the standard variation dataset of NA12878, but for more applications, more reference variation datasets from different populations are required^10^. Till now, there have been several Asian genomes publicly available from individuals of Chinese^11^, Korean^12^ and Pakistani^13^ descent. However, most of these genomes were sequenced only using Massive parallel sequencing (MPS, also known as next-generation sequencing, NGS) platforms, and thus high confidence variations in complex regions might not be resolved. For example, targeted DNA-HiSeq identified 1,281 SNVs in 193 genes in the Asian reference sample YH which were not detected in the original study^14^. These 193 genes were found to be probably associated with hereditary diseases with higher incidences in the Chinese population, also indicating the necessity of high-quality reference genomes in addition to NA12878^13^. It is now apparent that the combination of long reads, short reads, and co-barcoding read sequencing is required to fully characterize the variations in human genomes, and especially to establish high confidence variation dataset^15^. Herein, we established an Asian reference genome with genome-wide high confidence SNVs and indels by combining diverse sequencing platforms with short- and long- read, which could be an approach to mitigate the influences caused by systematic sequencing bias of different platforms.

## Results

### Sequencing and quality control

**Figure 1:**
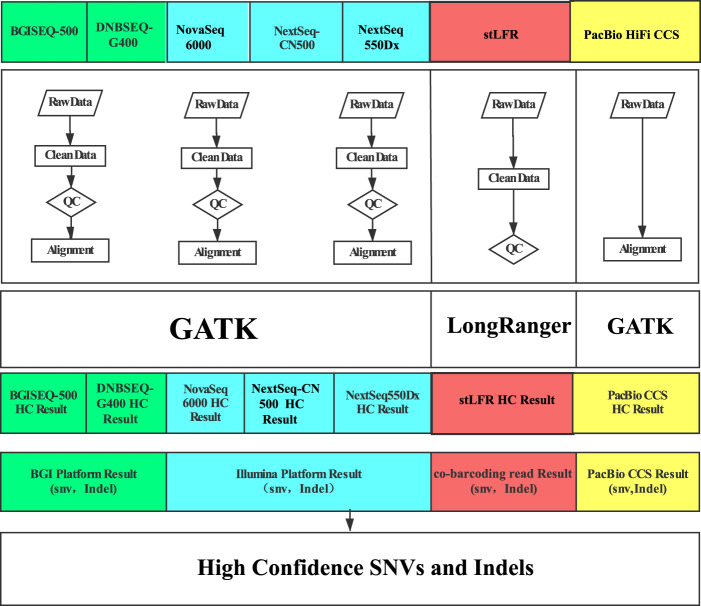
Overview of variation calling pipeline. The major steps included data filtering, alignment, variation calling, and integrated analysis.

To develop a representative high confidence variation dataset of Asian origin, we recruited a health Han Chinese adult male from Beijing, China (Research ethics ID: XHEC-C-2019-086, HJ). With the blood sample from the recruited individual, we constructed a cell line and after the fourth generation of the subculture, we finally obtained a stabilized cell line. We then extracted DNA from the stabilized cell line in a single batch and the extracted DNA were sequenced using five frequently-used massively parallel sequencing (MPS) short-read sequencing platforms (BGISEQ-500, DNBSEQ-G400, NextSeq-CN500, NextSeq550Dx and NovaSeq6000; three technical replicates for each of these platforms). We further applied single tube long fragment read (stLFR)^16^ technology on DNBSEQ-G400, and single-molecule real-time circular consensus sequencing (HiFi CCS) long-read^17^ on PacBio Sequel II to obtain long reads (synthetic long reads for stLFR). After data filtering (Figure 1), we obtained 3.56 Tb high-quality MPS sequencing data for this cell line in total. For the ordinary MPS data (short insert size libraries), we obtained an average coverage of 86.58× from each sequencing library on two BGI sequencers (2 × 100 bp), and 60.07× from each sequencing library on three Illumina sequencers (2 × 150 bp). We obtained 250.78 Gb (~83.02×) stLFR data with the average molecular length to be 11.7 kb, and 77.23 Gb (~24.4×) PacBio HiFi CCS data with an average read length of 12.1 kb. For the ordinary MPS data, ~99.88% of the filtered reads could be mapped to the human reference genome (hs37d5), resulting in a coverage of ~99.92%. Among these mapped reads, 85.75% can be uniquely mapped. For the stLFR data, we aligned 98.98% of the filtered data to the reference genome, resulting in 98.86% coverage. For the CCS reads, all of them can be mapped to the reference genome using pbmm2^18^ and the genome coverage was 93.2% (Figure S1 and Table S1).

### SNV and indel detection using MPS data

**Figure 2:**
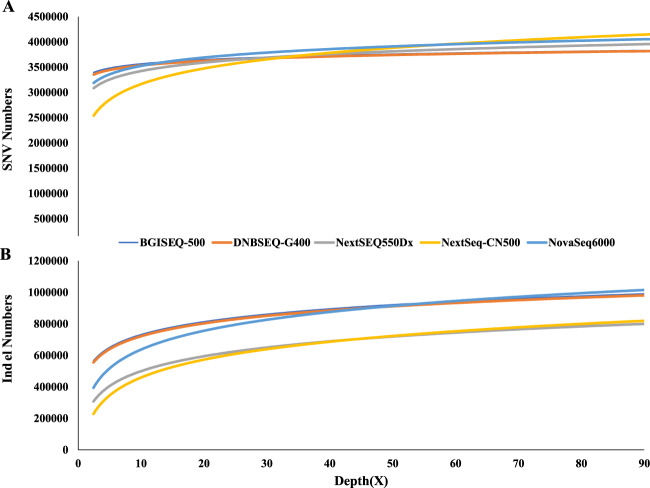
Saturation analysis. The relationship between SNVs(**A**)/indels(**B**) and depth, with the X axis for sequencing depth and the Y axis for the number of SNVs/indels detected.

To find the saturated sequencing depth of the different platforms for variation detection, we detected SNVs and indels in different sequencing depth fulfilled by randomly extracting from the alignment results. We found that 30× sequencing depth ensured consistency in the ratio of uniquely mapped reads (~99%) and the number of SNVs (~3.77 million) (Figure 2 and Figure S2). We noticed that the number of indels kept increasing as the read depth increased for short-read sequencing. To explore why the detected number of indels kept increasing beyond 30×, we compared the quality distribution of increased indels to those identified in 30× data. We found more low-quality indels were identified with more sequencing data. Thus, we thought the increased indels beyond 30× were more error prone, probably caused by accumulated sequencing errors(Figures S3 and S4).

**Figure 3:**
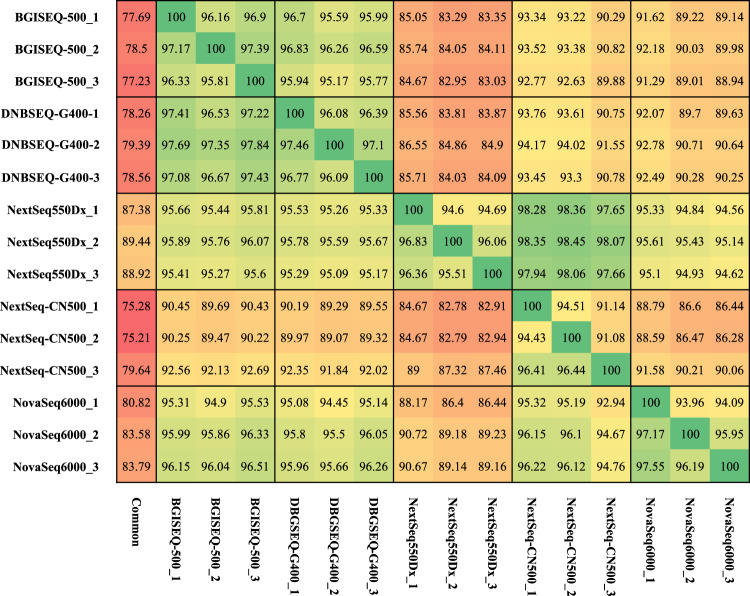
Blind zones by MPS in each sequencing platform.

We then evaluated the consistency of variations identified through the ordinary MPS data from the two different sequencing platforms (BGI and Illumina). Combining the replicates within platforms, we obtained 3,603,066 and 3,529,989 SNVs based on the data from BGI and Illumina platforms, respectively. We compared these two sets of SNVs to find 3,484,189 common SNVs (95.49%), 118,877 BGI platform-specific SNVs (3.26%) and 45,800 (1.25%) Illumina platform-specific SNVs (Figure S5). Nevertheless, despite the relatively high sequencing depth (~30×), ~44.41 Mb of the genome with 33.62 Mb of which to be located on chromosomes, could not be covered by single short-reads sequencing experiment (Figure3, Table S2,Table S3). These regions (here to be called “blind zones”) formed 51,612 blocks, with an average length of 860.55 bp, which possible composed by the specific Asian sequences and the regions recalcitrant to short-read MPS sequencing^19^. We aligned these blind zones against the YH reference genome and found about 28.19 Mb (~84.41%) sequences can be unambiguously matched, suggesting these 28.19 Mb blind zones were probably caused by the limitation of short MPS reads and the remaining blind zones might be the different regions between the two reference genomes. Interestingly, 73.3% and 68.53% of these blind zones were covered by stLFR and CCS reads (Table S4). Except for blind zones, we defined the remaining regions to be UMRs (uniquely mapped regions). We next wished to characterize SNVs and indels in the HJ cell line sequencing data that could not be mapped to the Caucasian reference genome, even with long-read sequencing data.

### Accessibility of SNVs and indels in blind zones

Using the stLFR data and the CCS data, we detected 3.87 M and 3.80 M SNVs, along with 822 K and 797 K indels, respectively (Table S1). Among these variations, we found a total of 74.7 K SNVs and 23.4 K indels were supported by both stLFR and CCS data but not detected using the ordinary MPS data. Those variations might be difficult to be identified through traditional whole-genome sequencing-based on short insert size libraries, and they can only be identified through long-read sequencing. We found these variations to affecting genes enriched in the gene ontology (GO) categories of olfactory receptor activity, IgG binding, transmembrane signaling receptor activity, G protein-coupled receptor activity, molecular transducer, and signaling receptor activity pathways. Among these 74.7 K SNVs, ~7.9% (5,913/74,700) SNVs located in blind zones, with 969 novel SNVs which were not included in the current databases of dbSNP and 1000 Genome database. Most of these novel SNVs located in the non-coding regions, with six of them in the coding genes. For instance, the gene LILRB3, which is associated with the diseases of Takayasu Arteritis and Anencephaly^20,21^, harbored such a novel nonsynonymous SNV.

We then used the 1000 Genomes database to assess the frequency of these 5,913 SNVs located in blind zones, classifying them into rare and common SNVs. We calculated the proportion of rare and common SNVs of the 301 Chinese dataset, the 504 East Asian dataset and the entire 1000 Genomes dataset. For the Chinese dataset, there are 52.81% and 35.64% rare SNVs in UMRs and blind zones, respectively. In the Asian dataset, there are 62.38% and 42.97% rare SNVs in UMRs and blind zones, respectively. In the entire 1000 Genomes dataset, there are 83.24% and 67.27% rare SNVs in UMRs and blind zones, respectively. Surprisingly, we found the percentage of rare SNVs to be high in all three datasets, and the percentage of rare SNVs in blind zones are notably less than that of UMRs (Table S5). We speculated the possible reason is that all the SNVs of 1000 Genomes database located in blind zones were identifiable, but these SNVs are sparse and the majority of SNVs in blind zones could not be detected using normal WGS short reads. Thus, we compared the SNV density between blind zones and UMRs in three datasets. Interestingly, we found the SNV density of blind zones is far less than UMRs in all three datasets with >10 times (Table S6).

**Figure 4:**
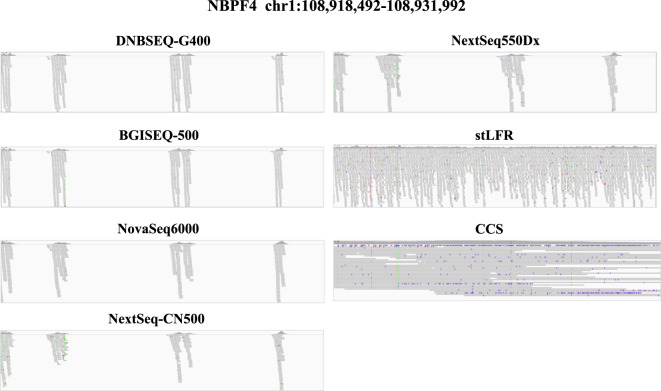
Depth and coverage of NBPF4 gene in blind zones.

In the blind zones, MPS is difficult to fully cover due to its read length, which may lead to false negatives of mutations, but stLFR and CCS perform well. Complex genes are hard to be covered by MPS platforms, while linked-reads method and long-reads sequences platforms do well in detecting the regions. For example, IGV shows a typical gene NBPF4^22^, who is a member of the neuroblastoma breakpoint gene family (NBPF) which consists of dozens of recently duplicated genes primarily located in segmental duplications on human chromosome 1 (Figure 4). Another gene is NAIP^23,24^ which is part of a 500 kb reverse replication on chromosome 5q13, contains at least four repeated elements and genes, and making it easy to rearrange and delete. The repeatability and complexity of the sequences also make it difficult to determine the organization of this genomic region. It is thought that this gene, modifier of spinal muscular atrophy, is a mutation in a neighboring gene SMN1. Variations detected on NAIP for MPS platform are relatively small and nearly included in linked-reads and long-reads platforms (Figure S6). In addition to the genes mentioned above, there is XAGE2 (Figure S7), and other genes.

### Comparison of different sequencing technologies and other variation benchmarks

**Figure 5:**
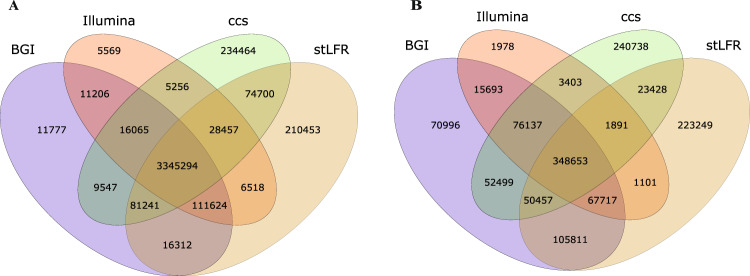
Consistency analysis: BGI regular MPS platforms, Illumina regular MPS platforms, linked-reads library, and PacBio CCS mode SNV(**A**) and indel(**B**) consistency analysis.

**Figure 6:**
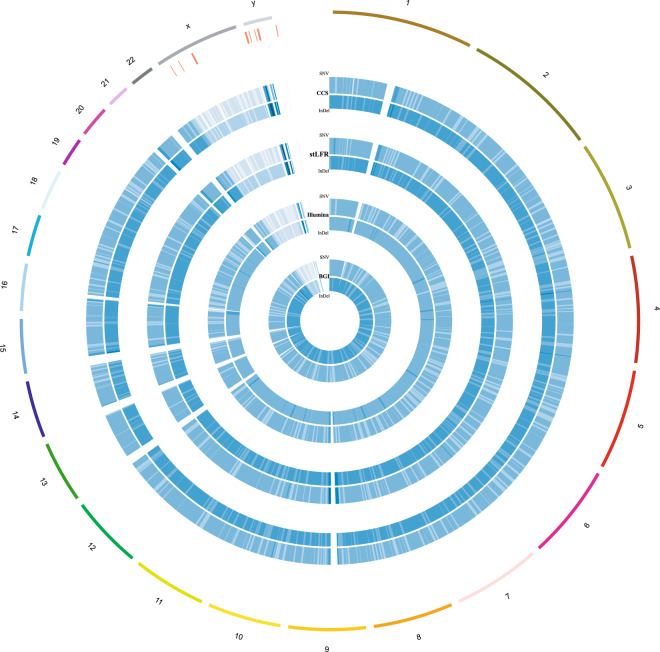
Density maps of SNV and indel variations normalized with Chinese population in 1000 Genome project. From inside to outside circles are DNBSEQ-MPS, Illumina-MPS, stLFR and Pacbio CCS respectively, and the last but one contains several lines, which means Chinese population failed in those regions detection while our data set contains variations here. Window =1 Mb, Inside and outside are indel and SNV.

Uniquely mapped regions (UMRs) were the exact opposite of blind zones which were in the non-N reference genome and easily mapping. In the UMRs, 3,345,294 SNVs and 384,653 indels could be detected by all seven sequencing methods (Figures 5 and 6). There were 234.46 K specific SNVs and 240.74 K specific indels using CCS data, 210.45 K and 223.25 K using stLFR, 11.78 K and 71.00 K using DNBSEQ-MPS, as well as 5.57 K and 1.98 K using Illumina-MPS (Figures 5 and 6). We compared the SNV quality distribution between specific SNVs and whole SNVs found that the quality of the majority of specific SNVs were lower than whole SNVs, likely stemming from sequencing method bias. Interestingly, CCS and stLFR consistently resulted in high-quality variant calls (Figure S8).

We finally identified 3.35 M SNVs and 348.65 K small indels of the HJ cell line by integrating all 17 data sets of seven platforms. In order to identify genetic variants of clinical significance, we annotated HJ cell line special variants against the ClinVar^25^ database. 4,404 variants (4,256 SNVs and 148 indels) in the HJ cell line were documented in ClinVar, including 37 variants that were classified as ‘pathogenic’. Among the data set, 52,026 SNVs and 18,148 indels had minor allele frequency (MAF) 0.01 in the 1000 Genomes Project of the Asian population, and 1.36 M SNVs were absent from the YH dataset. Comparing to NA12878 variant sets, 1.91 M SNVs and 176.72 K small indels were shared by the HJ cell line and NA12878. We also found 625.17 K SNVs shared by both the YH dataset and the HJ cell line dataset, suggesting these SNVs might be Asian only. We compared the characters of specific variations of HJ cell line, NA12878 and YH dataset, such as homozygous or heterozygous ratio, and found they showed a similar distribution in dbSNP, 1000 Genomes database and genomic regions (Table 1).

**Table 1:** Annotation of HJ, YH and NA12878 SNVs.

Sample	HJ	YH	NA12878
Total	3,345,294	3,072,912	3,259,653
dbSNP (%)	99.29	87.13	99.89
1000genomes (%)	98.28	95.93	98.68
Novel (%)	0.01	12.87	0.10
Homozygous	1,492,029	1,352,822	1,289,007
Heterozygous	1,853,265	1,720,090	1,970,646
Intronic	1,366,626	1,256,586	1,344,882
5′ UTRs	4,306	3,871	4,207
3′ UTRs	22,396	22,182	21,248
Upstream	47,789	43,612	44,056
Downstream	47,217	43,627	43,574
Intergenic	1,827,269	1,674,057	1,775,196
Ti/Tv	2.1	2.01	2.1

### Haplotype phasing small variants

Human genomes are diploid, with chromosome pairs from each parent. However, most paired-end reads cannot assign variants to a particular chromosome, resulting in a combined haplotype (genotype)^26^. Haplotype information is very useful for the identification of genetic variants associated with human diseases. Haplotypes can not be directly observed from the short-read sequencing except linked-reads but could directly observed using the long-read sequencing^27,28^. The popular MPS sequencing technology is all about shuffling sequences together for sequencing. We cannot directly distinguish which of these sequences are the parent source,but only after phasing we can make this distinction. Phasing is strongly correlated with the functional interpretation of genetic variation. Therefore, due to the BGI and Illumina short sequence reads generated from short-insert libraries, we using long-range information from PacBio HiFi CCS and stLFR data to phasing, 99.63% and 99.91% of heterozygous SNVs could be phased into 19,584 and 1,262 blocks, respectively. Of these, 1.96 M were shared, with a phasing N50 of more than 11.26 M and 388.5K. What’s more, some of the chromosomes (such as Chr5 and Chr6) were almost completely phased (Table 2). According to the results of phasing, stLFR data performed better, it showed that the long-range reads may a good choice in the phasing process.

**Table 2:** Haplotype phasing small variants.

Chr	CCS			stLFR		
	Heterozygous	Phased SNV	Phased rate(%)	Heterozygous	Phased SNV	Phased rate(%)
1	169,906	169,174	99.57	172,790	172,682	99.94
2	167,518	166,806	99.57	103,486	103,435	99.95
3	143,618	142,968	99.55	101,126	101,070	99.94
4	151,585	151,033	99.64	102,317	102,274	99.96
5	128,296	127,772	99.59	75,908	75,874	99.96
6	131,798	131,325	99.64	70,091	70,064	99.96
7	123,689	123,253	99.65	68,411	68,379	99.95
8	120,782	120,391	99.68	72,564	72,536	99.96
9	94,946	94,653	99.69	53,789	53,762	99.95
10	99,256	98,894	99.64	60,279	60,254	99.96
11	100,822	100,465	99.65	49,744	49,724	99.96
12	101,519	101,168	99.65	172,818	172,720	99.94
13	75,515	75,282	99.69	43,553	43,531	99.95
14	68,223	67,954	99.61	37,388	37,370	99.95
15	67,759	67,549	99.69	34,105	34,089	99.95
16	69,062	68,823	99.65	145,073	145,017	99.96
17	54,620	54,358	99.52	151,677	151,603	99.95
18	59,025	58,847	99.7	130,925	130,865	99.95
19	48,314	48,195	99.75	135,438	135,376	99.95
20	42,939	42,750	99.56	126,619	126,552	99.95
21	39,076	39,010	99.83	122,931	122,888	99.97
22	31,029	30,979	99.84	111,751	111,697	99.95
X	—	—	—	4,418	3,636	82.3
Y	—	—	—	4,444	4,354	97.97
Genome	2,089,297	2,081,649	99.63	2,151,645	2,149,752	99.91

## Discussion

Genome sequencing is an important part of precision medicine, widely used in the detection and diagnosis of various diseases, and brought potential benefits to patients. However, the MPS technologies also have some deficiencies, such as short reads and structural variation detection, especially the detection of variations in the blind zones. There is currently a lack of standard dataset that represents Asian populations due to ethnic differences. In this paper, a Han Chinese adult male was recruited and seven sequencing platforms were used to detect and integrate SNVs and indels. Finally, a total of 3.35 M high-quality SNVs were supported by seven methods, while co-barcoding read stLFR and long-read PacBio HiFi CCS resolved an additional 74.7 K SNVs, providing a comprehensive small variation benchmark of Asians. stLFR and CCS could be well supplemented and improved based on the MPS results. In addition, our study also identified 5,913 high-quality SNVs which located in the blind zones of MPS while supported by both stLFR and CCS long-read benefit from their long-range information. Our analysis revealed a number of unreported SNVs and small indels, supplied a completely high confidence standard small variant sets for further basic studies and precision medicine.

Many variation benchmark studies using cross-platforms, such as WGS or WES, were reported in recent years^9,11,29^. For the WES data, we all know, it just captures the exon regions, which are the small proportion of whole-genome sequences and many diseases caused by the mutations in the non-coding region were reported^30,31^. For the normal WGS sequencing data with short insert size, due to its limited alignment ability against the highly complex regions^32,33^, the complementation of the stLFR co-barcoding reads and CCS long reads used in our study fill the gap of the previous studies^16^.

Study limitations might arise as a consequence of the type of variant calling pipelines and parameters performed. As for the effect of process or parameters on the results, the different analysis pipeline, software and parameters will influence the accuracy and integrity of the variation calling results^34,35^. Several studies have conducted a detailed evaluation of 70 bioinformatics pipelines comprising the combination of 7 short-read aligners and 10 variant calling algorithms to process WGS samples. The results showed remarkable differences in the number of the variants were called by different pipelines and proved BWA + GATK is the optimal combination^35^. Besides, in our previous study, we also assessed multiple parameters of the WGS analysis strategy and finally adopted a similar pipeline^36^. Thus, in this study, we straightly used a similar analysis pipeline with the evaluated empirical parameters for MPS. For the analysis pipelines of stLFR reads, we used the long-range WGS pipeline to process stLFR reads for human germline variant calling and phasing^37^. Using a high accuracy pipeline to call variants of CCS long reads, which was also used in the previous study of human HG002/NA24385 with high precision and recall values of variant-calling^38^.

For the detection of SNVs in the blind zones of MPS technologies, we found two resources including the specific sequences of Asians and the inaccessible regions limited by short reads. The second resource could be remedied by long-range information technologies, such as CCS long reads and stLFR co-barcoding reads. The previous study reported that some additional regions that are now accessible with longer CCS reads include numerous medically-relevant genes which have been previously reported as recalcitrant to MPS sequencing^19,38^. With the powerful DNA co-barcoding strategy of stLFR, it enables analysis of regions which can be difficult for regular WGS. For example, SMN1 gene whose mutations are responsible for the genetic disorder SMA and its homolog SMN2 gene are extremely similar. Thus, this makes it impossible to analyze because it results in the ambiguous mapping of short reads, but stLFR successfully rescued those reads and properly mapped them using co-barcoding information. Taken together, we proposed the long-range information of stLFR and CCS data to help the SNV calling of some genomic regions more amenable to particular long-read technologies. Moreover, we noticed the variannts indentified by the two different long-read technologies were unique to its platform.So we checked all of the specific SNPs, including the sequencing depth, SNP calling quality, allele frequency and genome region. But we could not distinguish which result was accurate or not and we conclude that the ambitious result may be caused by different library construction or sequencing platforms.

In summary, MPS results will miss some mutations in the blind zones. By adding analysis results of stLFR and CCS platforms, standard data sets and high confidence regions that are considered relatively reliable can be obtained. This dataset can be well used for further study. In order to improve the data set, it may be necessary to add samples and analysis methods for integrated analysis.

## Methods

### Sample collection

This study was carried out in accordance with relevant guidelines and regulations, in line with the principles of the Helsinki declaration^39^ and was approved by the Instituted Review Board of Bioethics and Biosafety of BGI (BGI-IRB). In this experiment, cell line genomic DNA was prepared from the National Institutes for Food and Drug Control (NIFDC), and it contained 10 μg per tube. Used Qubit 3.0 to quantified the genomic DNA and agarose gel to make sure the genomic DNA molecular was not substantially degraded.

### Library and sequencing

Massive parallel sequencing (MPS) library construction was adopted by the normal MPS construction process. The difference between the BGI and Illumina platforms was that the former involved rolling amplification while the latter used PCR amplification technology. In particular, the DNBSEQ library protocol contained three steps: including making DNA nanoballs (DNBs), loading DNBs, and sequencing. Single tube long fragment read (stLFR) library construction physically breaked the DNA into fragments of about 50Kbps, and then Tn5 transposase was used for library construction, so that each identical fragment could bear the same barcode^16^, after the ligation step, PCR was performed and the library was ready to enter any standard MPS workflow.

Large-insert single-molecule real-time circular consensus sequencing (HiFi CCS) library preparation was conducted following the Pacific Biosciences recommended protocols^40^. In brief, a total of 60 μg genomic DNA was sheared to ~20 kb targeted size by using Covaris g-TUBEs (Covaris). Each shearing processed 10 μg input DNA and a total of 6 shearings were performed. The sheared genomic DNA was examined by Agilent 2100 Bioanalyzer DNA12000 Chip (Agilent Technologies) for size distribution and underwent DNA damage repair/end repair, blunt-end adaptor ligation followed by exonuclease digestion.

### MPS data preprocessing

Data filter: SOAPnuke (version 1.5.6) was used to pre-process the 15 MPS data by removing reads from raw data with (1) adaptor contaminations, (2) more than 10% low-quality bases (base quality <10), (3) more than 10% N bases.

Mapping and variant calling: All filtered reads were mapped to the human reference genome (hs37d5) using BWA 0.71.5^41^ (an in-house Apache Hadoop version) and removed duplication reads by Picard 1.23 (an in-house Apache Hadoop version). The Genome-Analysis-ToolKit (GATK) 2.3.9-lite^42^ (an in-house Apache Hadoop version) was used for variant calling from BAM files with HaplotypeCaller v2.3.9-lite.

### Saturation analysis of the MPS data

Picard (version 2.18.9) was used to randomly select BAM files from 10× to the maximum depth in a 10×-step for each MPS data. Next, MegaBOLT (version 1.15) was used for variant calling and then hard-filtering the SNVs with parameters of “QD < 2.0 | | FS > 60.0 | | MQ < 40.0 | | MQRankSum < −12.5 | | ReadPosRankSum < −8.0” and Indels with parameters of “QD < 2.0 | | FS > 200.0 | | ReadPosRankSum < −20.0”.

### Identification of the blind zones

For each MPS data, the read sequencing depth of the whole reference genome was calculated by GATK. First, N-bases in the reference genome were filtered out. Then, a non-N block or base in reference genome would be considered as uncovered for each MPS data if the sequencing depth was less than 5. Those non-N blocks or bases were considered homologous if they were uncovered by all 15 MPS data. Finally, all those non-N homologous blocks and bases were considered as blind zones. In the blind zones, using the short-read library and sequencing technologies might result in false-negative of variation calling. The remaining parts in the non-N reference genome except blind zones were defined as uniquely mapped regions (UMRs). In the UMRs, the sequencing reads were unambiguously mapped and used to perform SNVs and indels calling.

### Variants calling of the stLFR reads

The output files (FASTQ) of the linked-read sequencing method from the stLFR library and DNBSEQ-G400 sequencing platform, enabling the use of the 10X Genomics Long Ranger software after converting the stLFR barcodes to a Chromium compatible format. Firstly, we converted the stLFR barcodes to 10X Genomics barcodes used in-hourse Perl script. Then, we used SOAPnuke 1.5.6 to filter out low quality and adapter reads, and converted the data to 10X Genomics data format. Finally, clean reads were mapped and phased using the Long Ranger 2.1.2 wgs model. Briefly, de-multiplexed FASTQ files were de-duplicated, filtered,phased and SNVs/ indels were called. The SNV and indel information was parsed from the final VCF file using GATK SelectVariants.

### Data analysis of PacBio CCS reads

PacBio single-molecule real-time circular consensus sequencing (HiFi CCS) have low base error rates, providing both highly-accurate variant calls and long-range information needed to generate haplotypes. We used the pbmm2 (version 1.0.0) alignment tool to map reads which produced by PacBio HiFi CCS to the hs37d5 human reference genome, with the parameter “–preset CCS–sample HJ –sort”. GATK HaplotypeCaller was used to call SNVs and small indels. Different values of the HaplotypeCaller parameter “–PCR-indel-model” and VariantFiltration parameter “–filter-expression” were adapted, setting 60 as the minimum mapping quality, using allele-specific annotations and “–pcr_indel_model AGGRESSIVE”. SNVs and small indels were filtered using GATK VariantFiltration with "–filter_expression of AS_QD < 2.0". Longer read lengths improve the ability to phase variants, as tools like WhatsHap demonstrate for PacBio reads^40^.

### Haplotype phasing

Data from different technologies or BAM files for the same individual was used different tools for haplotype phasing. By using high-confidence variant calls which were standard SNVs VCF format and sorted BAM file, we adopted WhatsHap (version 0.18) phase and stats commands to phase and statistic variants for PacBio HiFi CCS platform data^43^. Linked reads were different from normal MPS short reads or long reads and required an extra step to link short reads together into co-barcoding molecules. HapCUT2 tools were suited for stLFR data to phasing, which designed for speed and accuracy across diverse sequencing technologies and good for diploid organisms phasing. The flowing three-steps were needed. First of all, the BAM file was converted to the compact fragment file format containing only haplotype-relevant information by extractHAIRS command. Next, we used LinkFragments command to link fragments into co-barcoded molecules. In the end, HAPCUT2 was used to assemble the fragment files into haplotype blocks^44^.

## Supplementary information

Supplementary information.

## Data Availability

The sequence data from this article can be found in the CNSA databases under the following accession numbers: CNP0000091.
