# Supplementary material for: An integrated Asian human SNV and indel benchmark established using multiple sequencing methods
Source: Sci Rep. 2020 Jun 17;10:9821. doi: 10.1038/s41598-020-66605-6 (PMC7300012; doi:10.1038/s41598-020-66605-6)
Supplement: Supplementary file 1 — Supplementary information. [file 41598_2020_66605_MOESM1_ESM.pdf]

An integrated Asian human SNV and indel benchmark established using multiple sequencing methods

Chuanfeng Huang[1, *], Libin Shao[2, *], Shoufang Qu[1, *], Junhua Rao[3, *], Tao Cheng[4], Zhisheng Cao[5], Sanyang Liu[6], Jie Hu[2], Xinming Liang[3], Ling Shang[4], Yangyi Chen[7], Zhikun Liang[8], Jiezhong Zhang[6], Peipei Chen[5], Donghong Luo[7], Anna Zhu[8], Ting Yu[1], Wenxin Zhang[1], Guangyi Fan[2,9,10], Fang Chen[3, †], Jie Huang[1, †]

1. National Institutes for food and drug Control (NIFDC), No.2, Tiantan Xili Dongcheng District, Beijing 10050, P. R. China
2. BGI-Qingdao, BGI-Shenzhen, Qingdao, Shandong, 266555, P. R. China
3. MGI, BGI-Shenzhen, Shenzhen, Guangdong, 518083, P. R. China
4. BerryGenomics Co., Ltd. Building #5, 4 Science Park Road, ZGC Life Science Park, Beijing, 102200, P. R. China
5. Tianjin Novogene Bioinformatic Technology Co., Ltd. Entrepreneurial Headquarters Base B07-B09, Wuqing Development Zone, Tianjin, 301700, P. R. China
6. Annoroad Gene Technology, Building B1, Yard 88, kechuang 6 Rd, Beijing Economic-Technological Development Area, Beijing, 102200, P. R. China
7. CapitalBio Genomics Co., Ltd., Building 11, GuanTai Biotechnology Cooperation Incubation Center, No.1, Taoyuan Road, Songshan Lake Hi-Tech Industrial Development Zone, Dongguan, Guangdong, 523808, P.R. China
8. Guangzhou Daruia Biotechnology Co. Ltd., 5 buildings No. 11 Nanxiang Third Road, Science City, Luogang District, Guangzhou, Guangdong, 510663, P.R. China
9. BGI-Shenzhen, Shenzhen, Guangdong, 518083, P.R. China
10. China National GeneBank, BGI-Shenzhen, Shenzhen Guangdong, 518120, P.R. China

*These authors contributed equally to this work.
†Correspondence authors: Fang Chen (fangchen@genomics.cn) and Jie Huang (jhuang5522 @126.com)

Supplementary Figure S1. Sequencing depth, duplicate and mapping coverage.
Supplementary Figure S2. Saturate analysis for SNV, Indel and coverage for MPS platforms.
Supplementary Figure S3. The Indel quality distribution along with sequencing depth increasing
Supplementary Figure S4. The Indel precision distribution along with sequencing depth increasing.
Supplementary Figure S5. Consistency of SNV from BGI and Illumina short sequence reads.
Supplementary Figure S6. IGV views NAIP gene for each platform.
Supplementary Figure S7. IGV views XAGE2 gene for each platform.
Supplementary Figure S8. All and platform unique SNV quality distribution for long fragment and short fragment platforms.

Supplementary Table S1-1. Data information of each platforms.
Supplementary Table S1-2. Data information of each platforms.
Supplementary Table S1-3. Data information of each platforms.
Supplementary Table S2. Statistics of blind zones.
Supplementary Table S3. Information of blind zones.
Supplementary Table S4. Phasing statistic for PacBio HiFi CCS and stLFR data.
Supplementary Table S5. Statistics of Allele frequency less than 1% in 1000 Genome database.
Supplementary Table S6. SNV and SNV density detected in blind zones and UMRs.

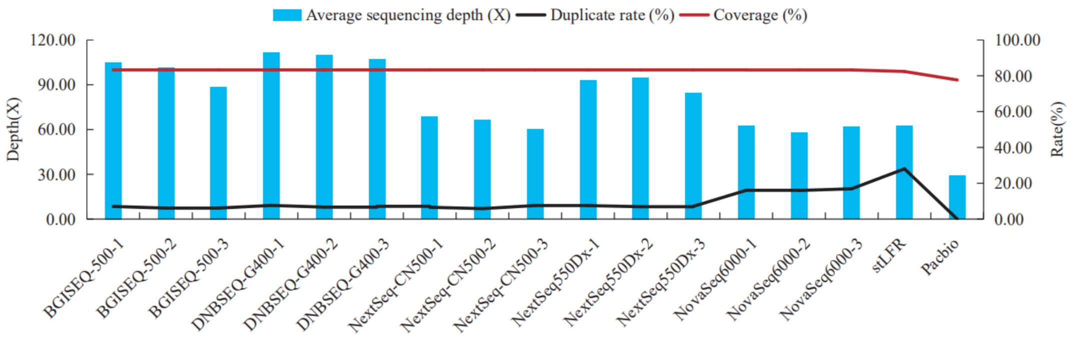

Supplementary Figure S1. Sequencing depth, duplicate and coverage. For each platform and technical replicate sample, histograms indicate the sequencing depth, duplicate rate and coverage are represented by black and red line respectively.

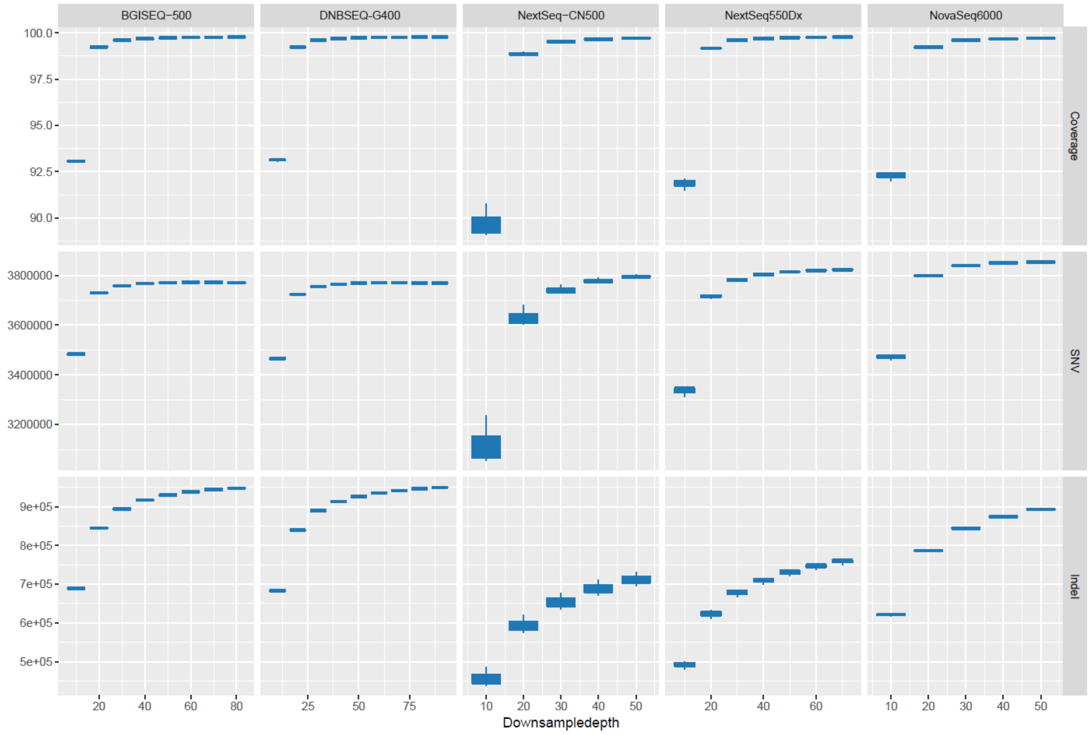

Supplementary Figure S2. Saturation analysis for SNV, Indel and coverage for MPS platforms. Each window shows average number for coverage, SNV and Indel Numbers correspond to Sequencing depth from BGISEQ-500, DNBSEQ-G400, NextSeq-CN500, NextSeq 550DX and NovaSeq6000 platforms.

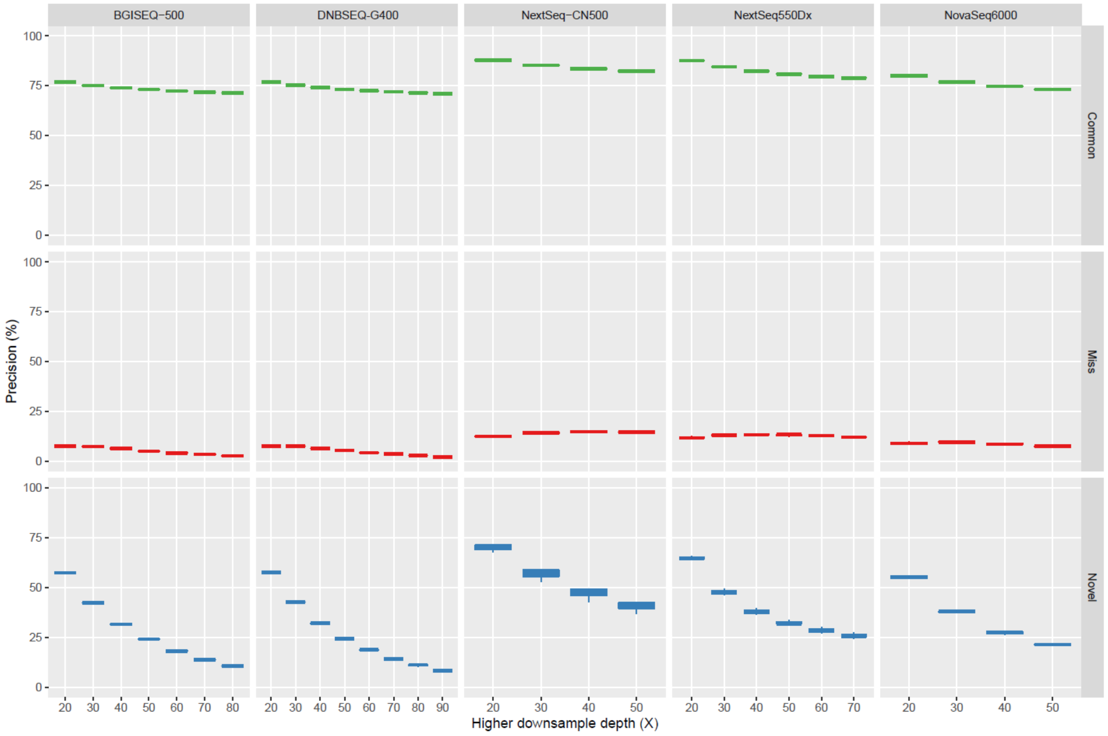

Supplementary Figure S3. The Indel quality distribution along wiht sequencing depth increasing. Three types Indel, common, missed and novel are shown in each round, and the dark line in the picture indicate the quality of final Indel set.

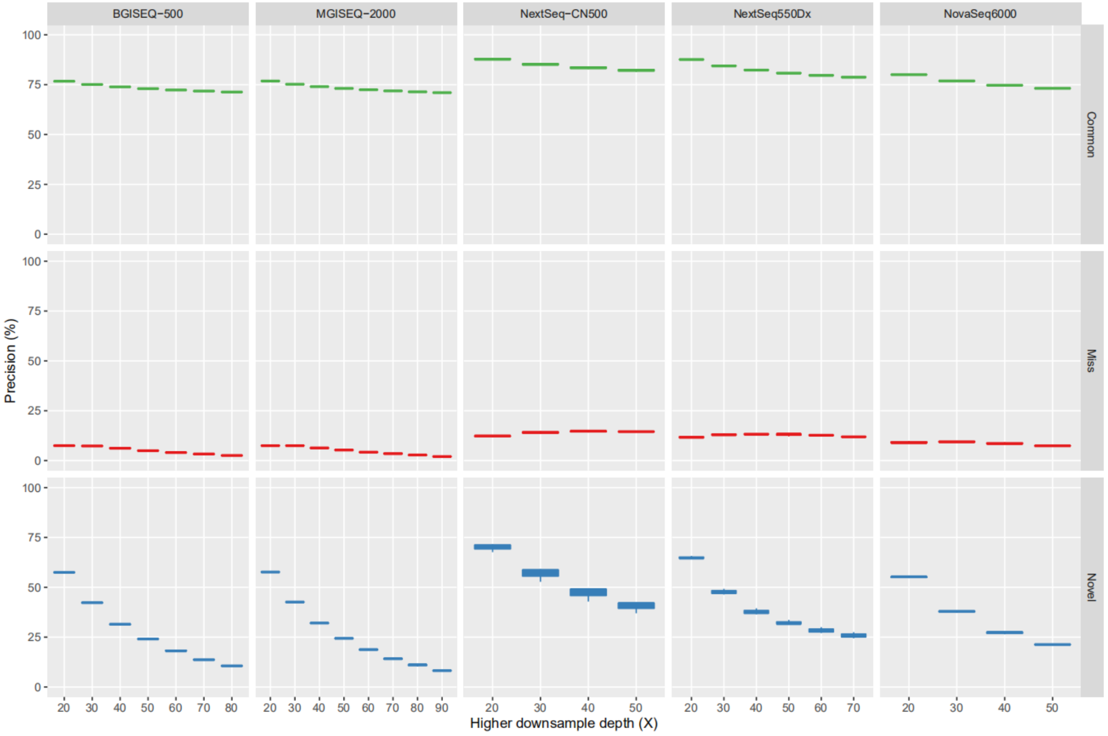

Supplementary Figure S4. The Indel precision distribution along with sequencing depth increasing. Common, missed and novel Indel classified in each round.

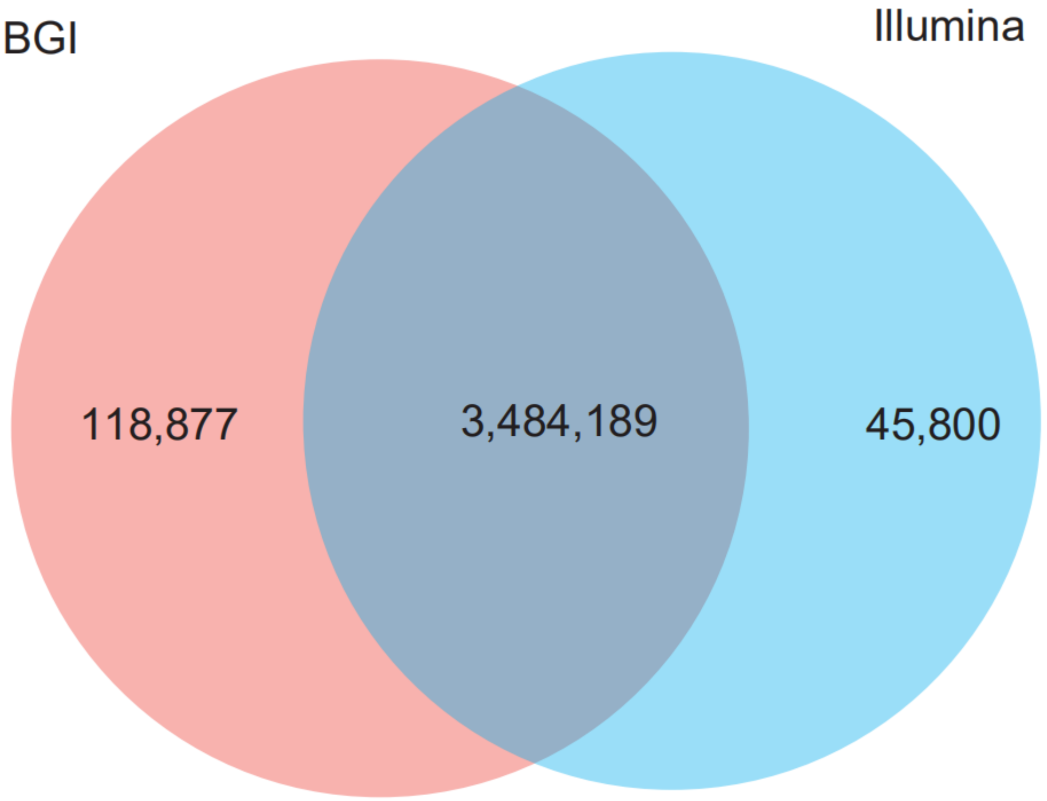

Supplementary Figure S5. Consistency of SNV from BGI and Illumina short sequence reads. SNV consistency, supported by all samples for BGI and Illumina platforms are shown.

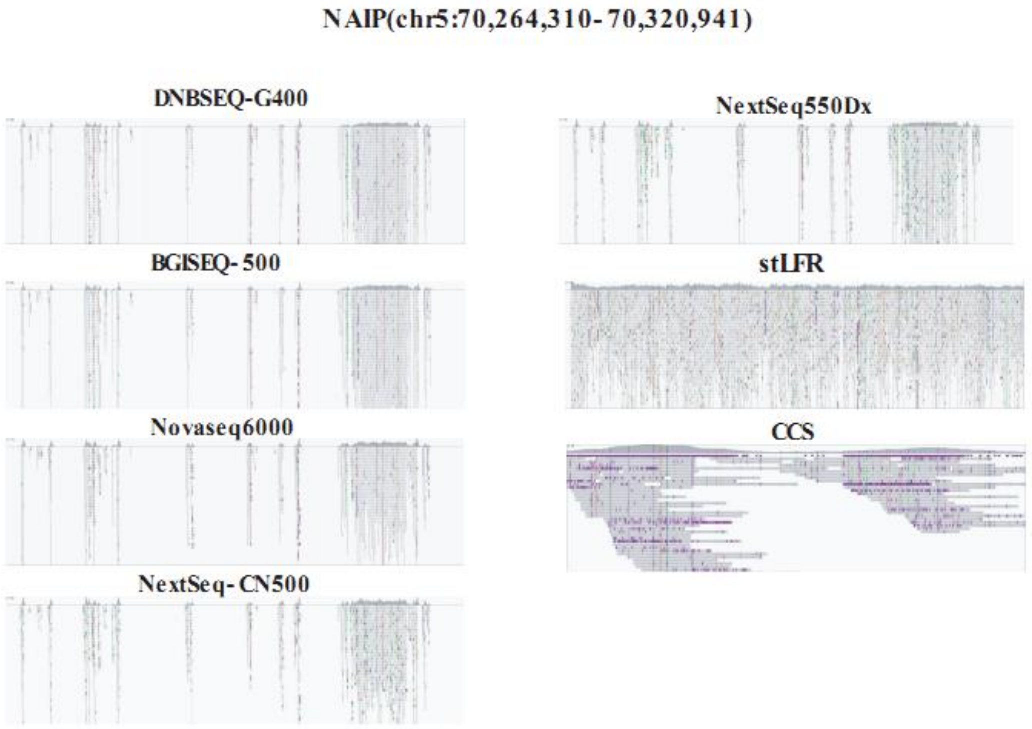

Supplementary Figure S6. IGV views NAIP gene for each platform.

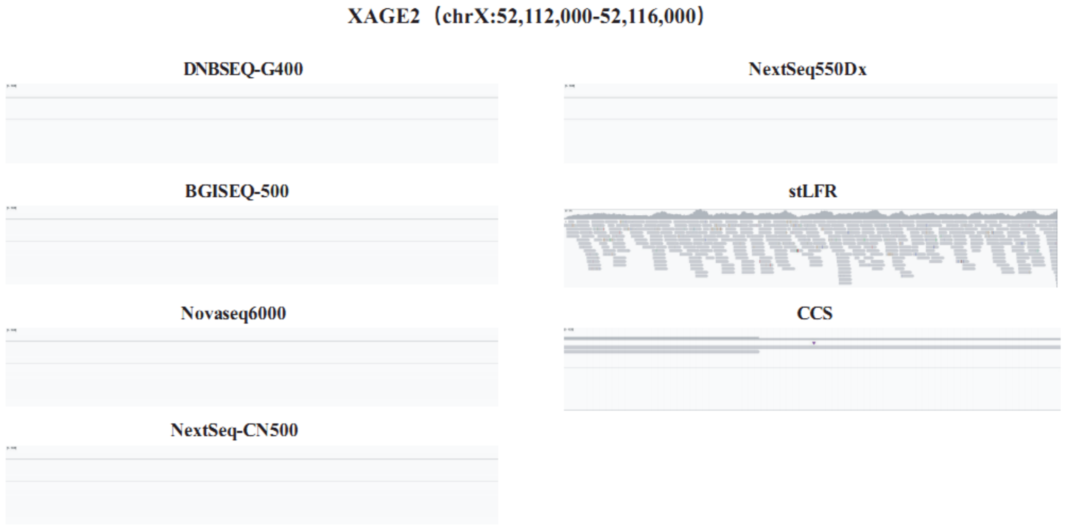

Supplementary Figure S7. IGV views XAGE2 gene for each platform.

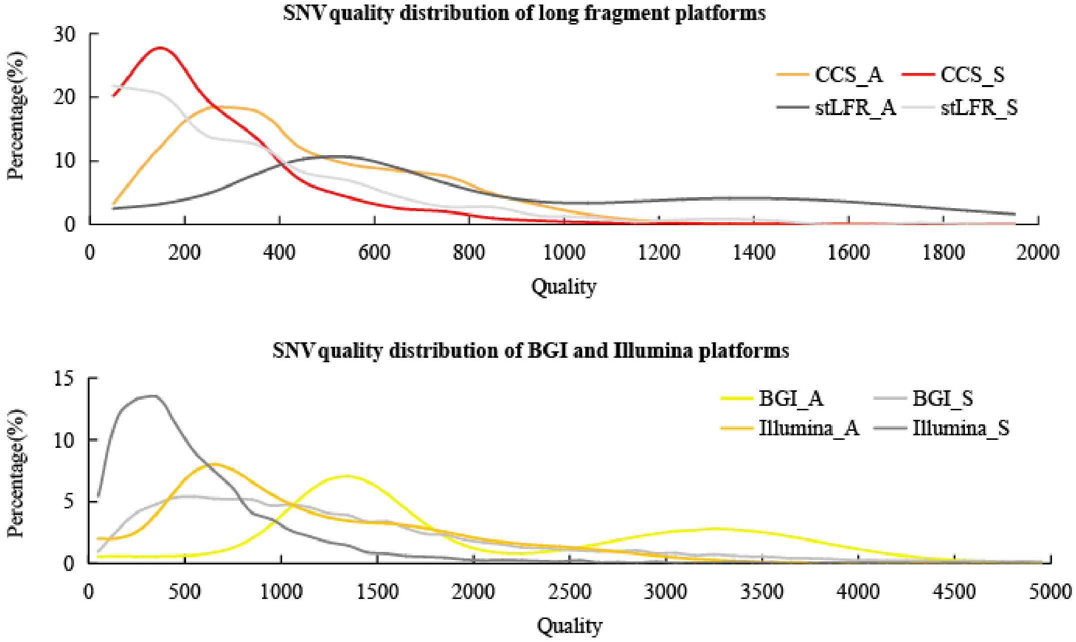

Supplementary Figure S8. All and platform unique SNV quality distribution for long fragment(A) and short fragment platforms(B). lines with tag "_A" indicates all SNV, "_S" means those unique.

Supplementary Table S1-1. Data information of each platforms.

	BGISEQ-500			DNBSEQ-G400		
	1	2	3	1	2	3
Clean reads	2,835,787,762.00	2,705,531,402.00	2,366,532,060.00	3,025,576,552.00	2,947,464,316.00	2,895,152,490.00
Clean bases (Mb)	283,578.78	270,553.14	236,653.21	302,557.66	294,746.43	289,515.25
Mapping rate (%)	99.97	99.95	99.96	99.98	99.98	99.98
Unique rate (%)	87.89	88.99	88.91	87.28	88.44	87.84
Duplicate rate (%)	8.47	7.37	7.39	9.16	8.02	8.57
Mismatch rate (%)	0.22	0.21	0.19	0.25	0.28	0.28
Average sequencing depth (X)	87.50	84.52	73.86	92.77	91.57	89.25
Coverage (%)	99.93	99.92	99.92	99.93	99.93	99.92
Coverage at least 4X (%)	99.88	99.88	99.88	99.89	99.89	99.88
Coverage at least 10X (%)	99.82	99.81	99.79	99.82	99.82	99.81
Coverage at least 20X (%)	99.67	99.65	99.59	99.69	99.69	99.65
SNV_number	3,731,347.00	3,722,674.00	3,712,120.00	3,685,587.00	3,696,424.00	3,707,393.00
indel_number	958,346.00	961,643.00	954,785.00	958,497.00	962,494.00	958,369.00

Supplementary Table S1-2. Data information of each platforms.

	NextSeq-CN500			NextSeq550Dx		
	1	2	3	1	2	3
Clean reads	1,312,748,450.00	1,257,851,333.00	1,151,358,700.00	1,736,721,953.00	1,751,990,056.00	1,561,010,158.00
Clean bases (Mb)	196,912.27	188,677.70	172,703.80	262,245.01	259,721.10	231,618.38
Mapping rate (%)	100.00	99.68	99.68	99.77	99.78	99.81
Unique rate (%)	86.94	87.89	86.32	87.57	88.45	88.19
Duplicate rate (%)	7.92	7.02	9.08	9.05	8.29	8.57
Mismatch rate (%)	0.55	0.55	0.52	0.43	0.43	0.41
Average sequencing depth (X)	56.97	55.10	50.22	77.61	79.02	70.30
Coverage (%)	99.93	99.93	99.93	99.93	99.93	99.93
Coverage at least 4X (%)	99.86	99.86	99.85	99.89	99.89	99.88
Coverage at least 10X (%)	99.67	99.67	99.64	99.81	99.81	99.79
Coverage at least 20X (%)	98.67	98.49	98.16	99.59	99.60	99.53
SNV_number	3,728,556.00	3,731,429.00	3,737,799.00	3,753,140.00	3,733,708.00	3,755,283.00
indel_number	728,748.00	711,141.00	739,453.00	781,824.00	794,458.00	784,894.00

Supplementary Table S1-3. Data information of each platforms.

	NovaSeq6000			co-barcoding	Pacbio
	1	2	3	stLFR	CCS
Clean reads	1,289,631,254.00	1,199,680,727.00	1,294,029,261.00	2,507,844,034.00	6,409,943.00

Clean bases (Mb)	193,444.69	179,952.11	194,104.39	250,784.40	77,229.46
Mapping rate (%)	99.88	99.91	99.92	98.86	100.00
Unique rate (%)	77.47	77.60	76.49	65.05	99.82
Duplicate rate (%)	19.28	19.16	20.31	33.66	0.00
Mismatch rate (%)	0.24	0.26	0.24	0.00	0.00
Average sequencing depth (X)	51.83	48.24	51.32	51.97	24.40
Coverage (%)	99.90	99.91	99.91	98.86	93.18
Coverage at least 4X (%)	99.84	99.84	99.84	98.72	92.83
Coverage at least 10X (%)	99.71	99.71	99.73	98.34	90.18
Coverage at least 20X (%)	98.69	98.23	98.68	96.61	62.55
SNV_number	3,751,598.00	3,716,984.00	3,734,579.00	3,874,599.00	3,795,024.00
indel_number	944,658.00	943,428.00	944,173.00	822,307.00	797,206.00

Supplementary Table S2. Statistics of blind zones.

Sample	of blind zone	Length of blind zone	Ratio of blind zone	Max length	Mean length	1-100bp Rate	>100bp Rate	Repeat rate	LowComplexity Rate	Mappability Rate
Common	51612	44414692	1.531361492	171822	860	43.88320546	56.11679454	28.06	3.55	63.58
BGISEQ-500-1	86531	56313296	1.941610064	191591	650	46.87915314	53.12084686	29.57	4.44	59.91
BGISEQ-500-2	88532	55809196	1.924229344	187034	630	48.99471378	51.00528622	29.61	4.55	59.69
BGISEQ-500-3	91721	56667668	1.953828355	187034	617	49.5698913	50.4301087	29.7	4.57	59.36
DNBSEQ-G400-1	84899	55945722	1.928936585	187034	658	46.66839421	53.33160579	29.52	4.38	60.12
DNBSEQ-G400-2	86376	55242444	1.904688464	186504	639	48.74270631	51.25729369	29.47	4.45	60.07
DNBSEQ-G400-3	87463	55712134	1.920882771	186480	636	48.52223226	51.47776774	29.74	4.58	59.72
NextSeq550Dx-1	83478	50647655	1.746266045	191340	606	57.32887707	42.67112293	28.18	4.78	60.51
NextSeq550Dx-2	82407	49566962	1.709005139	186428	601	58.15646729	41.84353271	28.06	4.85	60.39
NextSeq550Dx-3	89348	49892509	1.720229581	191283	558	61.03662085	38.96337915	28.07	4.93	60.01
NextSeq-CN500-1	114603	58055997	2.001696155	191434	506	59.23754177	40.76245823	28.96	5.58	58.48
NextSeq-CN500-2	113902	58131009	2.004282472	191558	510	59.32029288	40.67970712	28.91	5.44	58.45
NextSeq-CN500-3	103920	55262351	1.905374832	191311	531	60.31947652	39.68052348	28.62	5.63	58.71
NovaSeq6000-1	66490	54766189	1.888267804	186495	823	38.23432095	61.76567905	29.29	4.31	59.78
NovaSeq6000-2	65357	53158741	1.832845063	187034	813	41.36358768	58.63641232	29.05	4.22	59.73
NovaSeq6000-3	64218	53005900	1.827575301	186471	825	40.68018313	59.31981687	29.12	4.32	59.77

Supplementary Table S3. Information of blind zones.

Chrom	Blind zones length	Chrom length	Ratio(%)
1	3,060,143	249,250,621	1.23
2	1,748,941	243,199,373	0.72

3	457,106	198,022,430	0.23
4	704,697	191,154,276	0.37
5	1,743,794	180,915,260	0.96
6	566,981	171,115,067	0.33
7	1,624,082	159,138,663	1.02
8	1,082,236	146,364,022	0.74
9	5,077,275	141,213,431	3.60
10	1,799,872	135,534,747	1.33
11	560,946	135,006,516	0.42
12	334,952	133,851,895	0.25
13	146,952	115,169,878	0.13
14	250,485	107,349,540	0.23
15	1,498,743	102,531,392	1.46
16	1,441,022	90,354,753	1.59
17	815,769	81,195,210	1.00
18	93,484	78,077,248	0.12
19	410,028	59,128,983	0.69
20	218,335	63,025,520	0.35
21	410,743	48,129,895	0.85
22	682,048	51,304,566	1.33
X	2,959,833	155,270,560	1.91
Y	5,927,949	59,373,566	9.98

Supplementary Table S4. Phasing statistic for PacBio HiFi CCS and stLFR data.

	CCS	stLFR
Length(bp)	30403236	32519955
Percentage (%)	68.53	73.3
UTR3	61	155
UTR5	13	44
Downstream	348	421
exonic	397	441
Exonic;Splicing	1	1
Intergenic	16480	29512
NcRNA_intronic	5220	6489
NcRNA_exonic	416	517
NcRNA_intronic	3100	5866
NcRNA_splicing	2	8
NcRNA_splicing	1	3
Upstream	471	448
Upstream;Downstream	14	7

Supplementary Table S5. Statistics of Allele frequency less than 1% in 1000 Genome database.

Population	Blind zones (%)	UMRs (%)
Chinese	35.64	52.81
Asian	42.97	62.38
1000 Genome	67.27	83.24

Supplementary Table S6. SNV and SNV density detected in blind zones and UMRs.

Region	Type	Chinese	East Asian	1000 Genome
Blind zones	SNV	21,266	24,453	59,285
	SNV/Kb	0.596	0.685	1.661
UMRs	SNV	19,277,139	24,409,408	84,742,595
	SNV/Kb	6.3	7.977	27.694
